# Opportunities and Challenges for Next-Generation Cementitious Materials Under Rising Demand

**DOI:** 10.3390/ma19204270

**Published:** 2026-10-09

**Authors:** Anastasia N. Aday, Fabian B. Rodriguez, John T. Kevern

**Affiliations:** Energy Security and Systems Innovation Directorate, National Laboratory of the Rockies, 15013 Denver West Parkway, Golden, CO 80401, USA; ana.aday@nlr.gov (A.N.A.); fabian.rodriguez@nlr.gov (F.B.R.)

**Keywords:** next-generation cementitious materials, supplementary cementitious materials (SCMs), industrial waste byproducts, reclaimed ash, limestone calcined clay cement (LC3), biocements, reactivity assessment, scale-up implementation

## Abstract

**Highlights:**

• U.S. cement and concrete supply is organized around a two-tier readiness framework, readily deployable options and emerging concepts that could potentially contribute to the market consistently up to 2035–2040.
• Feedstock volume alone does not determine deployment readiness, resulting in a qualitative readiness assessment for the next-generation of cementitious materials.
• Reactivity testing, long-term durability evidence, supply-chain readiness, and formal qualification pathways function as an interdependent sequence for commercial deployment.
• Coordinated progress across researchers, standards bodies, and specifying agencies, rather than technical advancement alone, will determine how quickly these materials move from demonstration to dependable use.

**Abstract:**

The cement and concrete industry now has access to a wider variety of cementitious material options than at any point in its history, yet only a subset of these materials are deployed at scale. Current construction practices are evolving from traditional cements towards Portland limestone cements and blended systems such as Limestone Calcined Clays (LC^3^), alongside expanded use of established and emerging supplementary cementitious materials (SCMs) and alternative SCMs (ASCMs). Industrial by-products, including changing fly ash and slag streams, electric arc furnace slag, reclaimed ash, waste glass, mine tailings, and recycled concrete fines for enhanced carbonation are emerging as feedstock options to supplement demand for SCMs. This review provides a materials-centric synthesis of cementitious material options that could contribute to the U.S. cement and concrete supply through approximately 2035–2040. The materials are organized into two broad readiness classes: readily available materials and blended systems with existing feedstock streams and credible near-term adoption pathways, and emerging material concepts for which processing, logistics, or qualification frameworks are not yet sufficiently mature for industry-wide deployment. Readily available options include calcined clays, limestone calcined clay cement (LC^3^) systems, ground lightweight aggregate fines, and reclaimed ash, while emerging options include mine tailings, recycled concrete fines, electric arc furnace slag, waste glass pozzolans, carbonation-cured binders, and biocementation approaches. For each material family, the review evaluates availability, technical performance, and standards and adoption status, including feedstock volumes and geographic distribution, processing requirements, reported strength and durability performance, and compatibility with existing production and construction practices. Across both readiness classes, broader deployment will require robust reactivity and performance test methods, long-term durability and field-performance evidence, supply-chain readiness, and staged qualification and specification pathways. The review concludes by identifying practical priorities for researchers, standards bodies, and major buyers seeking to advance next-generation cementitious materials from laboratory evaluation and demonstration toward routine use.

## 1. Introduction

Global cement production has remained at a high level, while regional demand varies as infrastructure, housing, and industrial investment cycles are variable. In the U.S., relatively stable cement consumption has coincided with a growing reliance on imports, which increased by approximately 50–60% between 2019 and 2025. The U.S. Geological Survey (USGS) data indicate that this trend reflects several constraints in the domestic cement market, including closed or idle plants, underutilized capacity, ongoing plant upgrades, and the ready availability of imported cement [1]. Where upgrades are implemented, the primary goals are to expand capacity, improve distribution, or integrate alternative materials at different stages of the production process (for example, alternative fuels, blended cements, and enhanced grinding capabilities for slag cements), thereby reducing the energy intensity of clinker production and improving overall manufacturing efficiency and capacity [2]. Cement manufacture is energy intensive. In 2022, global cement production was approximately 4.16 Gt, with an average clinker-to-cement ratio of 0.71 and kiln thermal energy intensity of about 3.6 GJ per tonne of clinker [3]. To improve energy efficiency and reduce production costs while meeting rising demand, the cement industry has pursued clinker reduction strategies, primarily through increased use of supplementary cementitious materials (SCMs), adoption of alternative fuels, and process optimization. However, SCM availability is tightening, as demand for fly ash and slag is projected to outpace supply as coal-fired power generation declines and steel production shifts from blast furnaces to electric arc furnaces [4,5]. Furthermore, this supply constraint is occurring against a backdrop of substantial private investment in next-generation cement and concrete technologies, with venture capital increasingly directed toward novel cement chemistries, cement reduction and carbon dioxide mineralization pathways, and digital solutions [6]. This scenario motivates the identification and validation of alternative cementitious materials (ASCMs) that can be produced at scale while maintaining performance and economic competitiveness.

In evaluating candidate SCMs, production volume and geographic distribution are central to assessing practical deployment potential. Materials must be available in sufficient quantities and at the right locations to contribute meaningfully to regional or national concrete supply, rather than only to small-scale or specialty applications. Ground-Granulated Blast Furnace Slag (GGBFS) and coal fly ash have historically met these criterion as high-volume, low-cost, highly reactive industrial byproducts, but their declining availability and consistency require alternatives with comparable or complementary production potential. Silica fume, although highly reactive, is generated in relatively small quantities as a byproduct of silicon and ferrosilicon production and therefore remains confined to specialty uses. Natural pozzolans, including volcanic materials and calcined clays, have demonstrated SCM performance in regional studies, although their contribution at scale depends on local geology and processing infrastructure [7,8]. Other industrial byproducts such as mine tailings, waste glass, and recycled concrete fines are generated in substantial quantities in specific regions but face technical, logistical, and regulatory barriers that currently limit their use as mainstream SCMs.

Understanding this production-volume landscape is essential for prioritizing research, development, and standardization efforts. Materials with limited availability may be attractive for targeted or regional applications but cannot materially address a national SCM supply gap and present limited motivation for pursuing standardization. Conversely, abundant materials with modest intrinsic reactivity may become viable through appropriate processing, beneficiation, or blending strategies. This review therefore focuses on material families that have both credible hydraulic or pozzolanic function in Portland-based systems and realistic potential to contribute at scale in the United States by approximately 2035–2040.

A second overarching challenge in transitioning from established SCMs to alternative materials is managing compositional and performance variability. Conventional fly ash and GGBFS, despite being industrial byproducts, typically exhibit relatively consistent properties within defined specification limits, enabling reliable concrete performance prediction and quality control. Alternative materials, particularly waste-derived streams and regionally sourced natural materials, often display greater heterogeneity due to variations in feedstock, processing conditions, and source location. This variability influences reactivity and strength development, water demand and workability, setting time, durability, and compatibility with chemical admixtures. Batch-to-batch consistency may be limited, necessitating robust testing protocols and, in some cases, batch qualification procedures. Addressing variability requires predictive relationships between material characteristics and concrete performance, performance-based specifications that accommodate controlled compositional ranges, and quality-control systems adapted to variable feedstocks. Mechanical and chemical modification methods including grinding, thermal activation, beneficiation, and blending provide additional routes to normalize properties and manage variability; these strategies are considered in detail in the material-specific sections that follow.

In this review, candidate materials are grouped by supply class and stage of commercialization rather than by chemistry alone. The discussion first examines materials that are already available or credibly near-term for the U.S. market, including calcined clays, LC^3^-type systems, ground lightweight aggregate fines, and reclaimed ash. Emerging options include mine tailings, recycled concrete fines, electric-arc furnace slag, waste glass pozzolans, carbonation-cured binders, and biocementation concepts. For each family, the review synthesizes information on availability, technical performance, and the status of standards and specifications that govern adoption and highlights key gaps that must be addressed for routine use in U.S. concrete construction.

## 2. Paper Organization/Methodology

This review is organized around material availability, deployment potential, and functional role in cement-based construction in the U.S. through approximately 2035–2040, rather than strictly by material chemistry or individual research results. The central question is which cementitious material, ASCMs, low-clinker blended cements, and emerging binder systems can supplement or partially replace Portland cement in the U.S. cement and concrete supply over that time frame, given the constraints on feedstock volumes, processing infrastructure, and specification standards. To address this question, materials are grouped into two broad classes: (i) readily available materials and blended systems, where feedstock streams already exist at scale and at least limited use or clear near-term adoption pathways are evident; and (ii) emerging material concepts, where feedstocks exist but processing, logistics, or qualification frameworks are not yet mature enough to support industry wide deployment.

**Figure 1:**
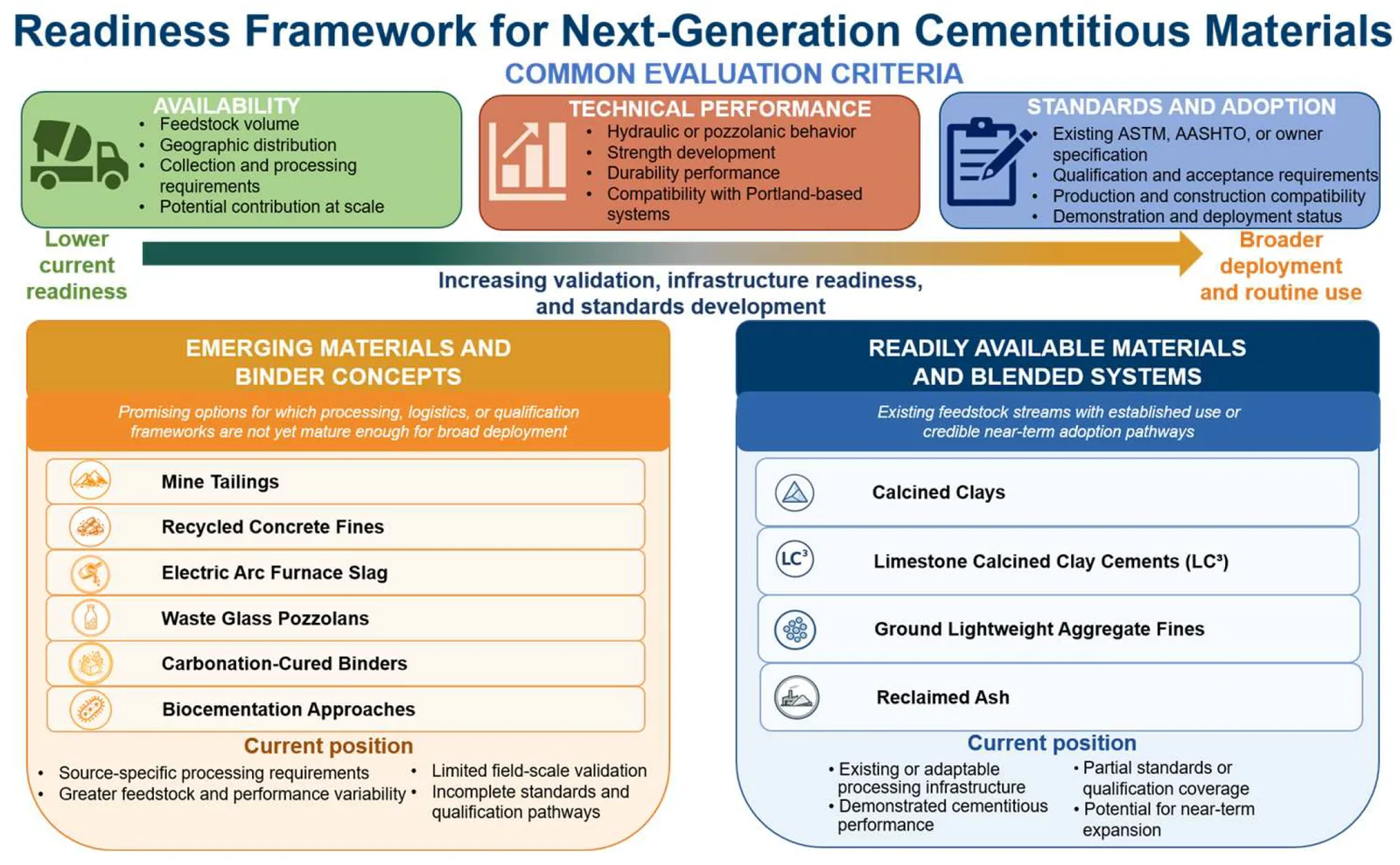
Readiness framework grouping next-generation cementitious materials into emerging and readily available categories based on availability, technical performance, and standards and adoption.

Within each class, individual materials families are discussed using a consistent three-part structure: availability, technical performance, and available standards for adoption. Availability focuses on order-of-magnitude deployable volumes and geographic distribution in the U.S., drawing on national statistics (e.g., USGS, industry associations, market reports) and recent syntheses of state DOT practices and emerging SCM resources. Technical performance summarizes reported hydraulic or pozzolanic behavior, effects on strength development and durability in Portland-based systems, and typical replacement levels identified in laboratory, pilot, and field studies. Available standards for adoption cover current ASTM, AASHTO, and state-level specifications and guidance, as well as ongoing work toward qualifying new sources and classes of SCMs and blended cements. The two readiness classes and the common evaluation criteria applied throughout this review are summarized in Figure 1.

The literature base for this synthesis was assembled through targeted searches of journal databases and agency reports, complemented by national-level surveys and syntheses on SCM use in transportation infrastructure. Priority was given to sources that (i) report quantitative information on U.S. or North American material volumes and usage patterns, (ii) evaluate the performance of alternative SCMs and blended cements in concrete, and (iii) document current specification and qualification practices by state DOTs and major owners. Scenario studies and adoption analyses for specific systems such as limestone-calcined clay cements (LC^3^) are used to inform potential deployment ranges where available.

The scope of this paper is intentionally limited to hydraulic and pozzolanic materials compatible with Portland-based binders and related calcium silicate or aluminosilicate systems. The discussion first examines material families and blended systems that are already available or credibly near-term for deployment in U.S. concrete, including clays (calcined clays, LC^3^-type systems, and ground lightweight aggregate fines) and reclaimed ash, and then turns to material families and concepts whose use remains emerging and non-commercial, such as electric-arc furnace slag, selected mine tailings, waste glass pozzolans, carbonation-cured binders based on hydraulic precursors, recycled concrete fines, and biocementation approaches.

## 3. Supply Readily Available

### 3.1. Calcined Clay, Limestone Calcined Clay Cements (LC3), Ground Lightweight Aggregate Fines (LWA)

Calcined clays, including metakaolin-grade materials and mixed clays, are among the most promising non-traditional SCM families from a supply perspective because suitable clay deposits are widespread and not tied to specific industrial processes. Kaolinitic and mixed clays occur in many U.S. regions, and additional clay-rich materials are present in industrial by-products such as certain paper sludges and oil-sand tailings [9,10,11]. Commercial metakaolin products are already available in the U.S. and are used at modest volumes in high-performance and durability-sensitive applications, and recent market analyses indicate growing interest in expanding calcined-clay SCM capacity through 2035 as part of broader clinker-substitution strategies [12,13]. As of May 2025, the Green Cement Technology Tracker identified four commercial U.S. calcined-clay facilities: Ash Grove’s Louisville facility in Nebraska and facilities operated by Burgess Pigment, Imerys, and KaMin in Georgia [14].

Limestone calcined clay cements (LC^3^) build on the same clay resource base but require plant-scale integration of calcination and blending. In principle, LC^3^ uses locally available kaolinitic or suitable mixed clays and widely available limestone, combined with Portland clinker and gypsum, to produce cements with clinker factors around 50% [15]. International experience shows that LC^3^ can be produced in existing cement plants by installing dedicated clay calciners and adjusting grinding and blending operations. Several U.S. demonstration and early commercial projects are now under way or in development as of 2025, including LC3 production by Roanoke Cement Company and Summit Materials (a subsidiary of Quikrete) [16,17,18,19]. While total U.S. LC^3^ tonnage remains small today, the combination of widespread clay deposits and existing limestone and clinker infrastructure suggest that LC^3^-type blends could reach multi-million-ton annual production if calcination capacity and specifications are developed in parallel. While LC3s components may pass their associated ASTM standards, large scale adoption by U.S. Department of Transportation (DOTs) has yet to be achieved. Increased use of LC3s in pilot demonstrations would serve to demonstrate their performance characteristics and further user confidence and acceptance in the future [20].

Ground lightweight aggregate (LWA) fines provide an additional clay-derived SCM pathway. Expanded shale, clay, and slate LWA is produced commercially in the U.S., and production generates fines that can potentially be recovered and milled; however, the recoverable volume of this by-product stream is not well quantified [21,22]. Studies have evaluated finely ground LWA at cement replacement levels of about 10–20%, with ~15% representing a commonly reported experimental level [23,24,25]. As interest grows in valorizing fines from aggregate and LWA production, these materials represent a modest but regionally meaningful additional clay-derived resource.

Calcined clays exhibit pozzolanic behavior when heated in the range of roughly 650–850 °C, where dehydroxylation of kaolinite and related clay minerals produces an amorphous aluminosilicate phase (metakaolin) with high reactivity in alkaline pore solutions. Metakaolin-grade materials typically improve early-age strength, reduce permeability, and enhance resistance to alkali–silica reaction and some forms of chemical attack when used at replacement levels of about 5–20% in Portland-based concretes [26,27]. Mixed clays with lower kaolinite contents can also function as SCMs after calcination, although reactivity depends strongly on mineralogy, calcination temperature and duration, and cooling conditions. Recent work has proposed reactivity indices and rapid tests (for example, based on heat release or bound water) to classify calcined clays as low, moderate, or high reactivity, highlighting the need for robust characterization frameworks [28,29,30].

LC^3^ formulations combine clinker, calcined clay, limestone, and gypsum in proportions that exploit the synergy between aluminates from the calcined clay and carbonates from the limestone. Experimental and pilot-scale studies indicate that LC^3^ blends with clinker contents around 50% and total calcined clay plus limestone contents around 50% can achieve mechanical properties comparable to or better than conventional Portland cement concretes at equivalent water–binder ratios, with particularly strong performance at later ages [15,31]. LC^3^ systems also show favorable durability characteristics, including reduced chloride ingress, improved resistance to alkali–silica reaction, and in many cases lower permeability, although mix-design adjustments to sulfate and alkali balance are important. Workability and setting behavior depend on the fineness and proportioning of the individual components; separate grinding and optimization of particle size distributions can mitigate challenges associated with intergrinding clinker, calcined clay, and limestone.

Finely ground LWA derived from expanded clay show pozzolanic reactions similar to other calcined clay materials, with performance depending on firing conditions, glassy phase content, and fineness [24]. When used to replace 15% of cement by mass, such fines gave 28-day compressive strengths approximately equal to the reference mix at 20 °C and 15% higher at 35 °C, but 10–15% lower at 5 °C, and have been reported to enhance resistance to chloride ingress, sulfate attack, alkali–aggregate reaction, and freeze–thaw cycling [32,33]. Pozzolanic activity generally increases with fineness, although the required grinding level depends on source mineralogy and firing history and adds processing cost. These materials therefore function as both pozzolanic SCMs and fine fillers, contributing to refined pore structure and improved durability in high-performance concretes [24,32,34]. Collectively, these clay-derived systems offer a technically robust basis for low-clinker cements and alternative SCMs, with adoption pathways considered in a later section of this review.

### 3.2. Reclaimed Ash

Coal fly ash is the particulate material collected from flue gases of coal-fired power plants and has been used as a supplementary cementitious material since the 1930s [35]. Fly ash has been valued globally for its pozzolanic reactivity, spherical particle morphology, and beneficial effects on fresh and hardened concrete properties. Typical fly ash replacement levels are 15–25% for Class F and 20–35% for Class C, while high-volume mixtures containing 50–70% fly ash have been demonstrated with high-range water reducers [36]. Coal fly ash is specified under ASTM C618, while natural pozzolans are addressed separately under ASTM C1945 [37,38]. However, fly ash supply is declining in many markets as coal-fired power plants are decommissioned in favor of natural gas and more energy-efficient sources. A 2020 U.S. market forecast projected fly ash production declining from 36.2 million short tons in 2018 to 30.8 million short tons in 2039 under its baseline scenario [5]. European markets face similar supply constraints [4]. Beyond volume reductions, the quality and consistency of available fly ash are also changing. Combustion of lower-grade coals, co-firing with biomass, installation of emissions control technologies, and operational changes at remaining power plants have altered fly ash chemical composition and physical properties [39]. These compositional shifts affect loss on ignition (LOI), carbon content, lime content, particle size distribution, and pozzolanic reactivity. However, the extent of these influences and their effects on concrete performance is not clearly determined [39,40]. This evolving supply landscape has motivated interest in reclaimed ash as a means of extending the usable coal-ash resource.

In response to tightening domestic supply, the fly ash industry has begun developing alternative sources and strategies for utilization in construction applications. Harvesting fly ash from legacy disposal ponds and landfills represents a significant potential resource. In 2017, 179 U.S. utility plants disposed of 20.2 million short tons of fly ash, while more than 1 billion tons of legacy fly ash have accumulated in landfills and surface impoundments [5,41]. Large-scale commercial harvesting operations are already supplying high-quality fly ash to concrete markets in South Carolina and Pennsylvania [42], guided by the recent approval of ASTM E3183-24 “ Standard Guide for Harvesting Coal Combustion Products Stored in Active and Inactive Storage Areas for Beneficial Use” [43]. These disposal-site totals represent an upper bound on potential feedstock rather than a realistic near-term supply. The fraction of reclaimed ash that will ultimately meet ASTM C618 specifications depends on site-specific conditions discussed below. Additionally, commercial beneficiation technologies, including moisture correction, chemical treatment, electrostatic separation, and carbon burn-out are being employed to improve ash quality and increase the portion of material suitable for use as SCM [42]. Despite these alternative sources, the combination of declining domestic production, quality variability, and the need for consistent high-performance materials creates an urgent need to identify and validate additional cementitious materials that can be produced at scale.

Reclaimed fly ash from legacy disposal ponds and landfills, combined with beneficiation technologies applied to both fresh and reclaimed material, offers a strategy to extend coal ash supply. Beneficiation processes include electrostatic separation to remove carbon particles (reducing LOI), grinding and air classification to remove coarse particles and improve fineness, chemical treatments to modify surface properties, and carbon burn-out through controlled thermal treatment [41]. As noted earlier, over 1 billion tons of ash materials exist in U.S. disposal facilities, with harvesting operations already active in multiple states [41,42]. Site-specific beneficiation can produce harvested fly ash meeting ASTM C618 requirements through processes such as drying, screening, grinding, separation, and thermal treatment [41,42]. However, challenges include significant variability in reclaimed ash quality depending on disposal history and weathering, energy and cost requirements for beneficiation processes, particularly thermal treatments,, need for case-by-case evaluation of reclaimed material properties, potential presence of contaminants from disposal site conditions, and regulatory considerations regarding ash removal from legacy sites [41,44]. Economic viability depends on local fly ash market prices and beneficiation costs.

Taken together, these factors position reclaimed ash as a technically credible extension of the traditional fly ash resource, but one whose broader deployment will hinge on consistent beneficiation practices, robust characterization, and appropriate qualification frameworks addressed elsewhere in this review.

## 4. Supply Emerging

### 4.1. Mine Tailings

Mine tailings represent a vast, geographically distributed resource with potential as cementitious feedstocks, particularly tailings from hard rock mining operations rich in aluminosilicate minerals. Suitable tailings typically contain quartz, feldspars, micas, and clay minerals in compositions amenable to pozzolanic or hydraulic activity when appropriately processed. Extraction for cementitious use involves retrieval from tailings storage facilities, with processing steps including drying (if wet-stored), screening to remove oversized particles, and, in many cases, grinding or thermal/mechanical activation to enhance reactivity. Global mine tailings production was estimated at more than 8 billion tonnes in 2016, with cumulative inventories of approximately 223 billion tonnes stored in 29,000–35,000 active, inactive, and abandoned tailings facilities worldwide [45,46]. In the United States, large volumes of tailings are present at active and abandoned sites, with particularly high concentrations associated with hard-rock mining in western regions, though robust national tonnage estimates remain uncertain and site-specific [47].

Because these totals aggregate highly heterogeneous deposits across variable ore types, mineral and heavy metal content, and site accessibility, they substantially overstate the volume of tailings that could realistically be qualified and transported for cementitious use in the near term. The technically and economically deployable fraction is likely a small percentage of the total inventory, with the realistically deployable fraction depending on site-specific characterization and processing decisions addressed later in this review.

From a performance perspective, a growing body of work has demonstrated that selected tailings can function as supplementary cementitious materials or aggregate replacements when appropriately characterized and activated. Tailings with sufficient amorphous content and favorable oxide composition (notably SiO_2_, Al_2_O_3_, Fe_2_O_3_, and CaO) have shown pozzolanic contributions to strength and durability when used at moderate replacement levels, while more inert crystalline tailings can act primarily as filler unless they undergo mechanical, thermal, or chemical activation to increase reactivity [48]. Performance varies substantially with tailings type and activation route. An iron-tailings/fly ash/GGBFS SCM blend used at 30% cement replacement reached 49 MPa at 28 days [49], while mechanochemically activated Pb–Zn sulfide tailings used at 20% replacement reached 40.85 MPa at 90 days compared with 38.15 MPa for the control [50]. These results demonstrate that selected tailings can contribute meaningfully to blended cementitious systems, although performance remains highly dependent on mineralogy, fineness, blend composition, and activation conditions.

Despite this technical potential, major drawbacks limit widespread adoption of mine tailings as cementitious materials. Compositional variability between ore types and even within a single deposit is extreme, making generic specifications difficult to apply. Many tailings contain sulfide minerals and trace metals that raise concerns about acid generation, leaching, and long-term environmental behavior, necessitating careful characterization and, in some cases, stabilization [51]. Regulatory complexities around mine-waste handling, ownership, liability, and transportation further constrain access to tailings as industrial feedstocks, and many large tailings facilities are located in remote areas far from major concrete markets, increasing logistics costs [52,53]. In practice, mechanical, thermal, or chemical activation may be required to achieve acceptable reactivity, adding processing cost and energy demand, so mine tailings are best viewed as a very large but technically and institutionally challenging emerging resource whose deployment will depend on site-specific projects, extensive characterization and thermal and/or mechanical processing before becoming a viable commodity in the concrete market.

### 4.2. Recycled Concrete Fines

Recycled concrete fines are the powder fraction generated during concrete crushing and processing, typically below 75 µm or 150 µm depending on the classification method used [54,55,56]. These fines contain partially hydrated cement paste, unreacted cement clinker, and small aggregate particles, and their composition depends strongly on the source concrete, including its mix design, age, and service history [55]. During recycled-aggregate production, the fine fraction can account for approximately 20–30% of the waste concrete processed [57]. Because construction and demolition waste is generated in large volumes and near major population centers, recycled concrete fines represent a geographically aligned but still underused cementitious feedstock. Fine material is generally not used as aggregate and is typically blended with natural sand or discarded, so much of the fines generated never re-enters the value chain and only a small portion currently serves any cementitious purpose [58].

In their untreated form, recycled concrete fines generally show limited hydraulic or pozzolanic activity, so their contribution in blended systems is often associated with filler effects, nucleation effects, and the presence of residual unhydrated cement phases [54]. Their performance can improve substantially after carbonation treatment. Accelerated carbonation converts calcium rich phases in the fines into calcium carbonate, refines microstructure, and can reduce porosity and water absorption under favorable processing conditions [55,59]. In one aggregate-reuse study, mortars with 70% sand replacement by carbonated recycled concrete fines developed about 20% higher 28-day compressive strength than mortars containing the same amount of non-carbonated fines [59]. Reviews also indicate that carbonation can create surface reactivity and promote hydration in blended systems, making recycled fines more attractive as a low carbon cementitious ingredient [56].

Despite this technical promise, several limitations currently prevent widespread deployment. Recycled concrete fines vary significantly with source material and processing conditions, which complicates prediction of reactivity and concrete performance [60]. Their high surface area and residual porosity can increase water demand, while contaminants from demolition and recycling operations, such as gypsum, masonry fragments, and organics, may adversely affect durability and consistency. Carbonation treatment requires dedicated processing and source-specific quality control, and further work is needed to define optimal treatment conditions, cement-replacement levels, and long-term field performance. For these reasons, recycled concrete fines are best viewed as a promising emerging resource that could link concrete recycling, carbon mineralization, and clinker reduction, but that still requires further technical validation before broad adoption.

### 4.3. Electric Arc Furnace (EAF) Slag

Slag availability and composition are intrinsically linked to ironmaking and steelmaking processes. Ground granulated blast furnace slag (GGBFS) is produced from blast furnace operations in integrated steel mills, where iron ore is reduced with coke and lime at high temperatures. Lime acts as flux and combines with silicates and oxides to form liquid slag, which floats on the molten iron due to its lower density. The molten slag is rapidly quenched with water to form a glassy material with latent hydraulic properties suitable for use as a cementitious binder, owing to its high content of amorphous silicates, aluminosilicates, and calcium that facilitate dissolution and participation in the hydration process [61]. In contrast, electric arc furnace (EAF) slag is generated during steel production from scrap metal recycling. EAF slag exhibits fundamentally different properties from GGBFS with respect to crystalline structure, mineral composition, and reactivity in cementitious systems; thus EAF slag is primarily used as aggregate rather than as an SCM [62], though recent research has explored beneficiation methods to enhance its suitability for concrete applications [63,64,65,66]. Integrated steel mills have the capacity to produce basic oxygen furnace (BOF) slag during steel refining and ladle slag during final processing stages. However, volume stability concerns due to free lime and magnesia content limit the use of BOF slag as a SCM, while ladle slag exhibits varying degrees of reactivity and similar volume instability issues [67,68,69,70].

The global steel industry is undergoing a structural transition from blast furnace-based production to EAF routes, driven by scrap availability and energy efficiency targets [71]. Global EAF share increased to 29.1% in 2024, up from 28.6% in 2023, resulting in approximately 548 million tonnes out of the 1.885 billion tonnes total global steel output [72,73]. The United States operates roughly 100 EAF mills and produces about 70% of its steel via the scrap-intensive EAF process. This shift has profound implications for slag supply. Although total steel production may remain stable or grow, the proportion of high-quality GGBFS suitable for concrete is declining as blast furnaces are decommissioned and replaced with electric-arc furnaces.

According to the U.S. Geological Survey (USGS), approximately 16 million tons of slag were sold in the United States in 2025, with granulated blast-furnace slag accounting for less than 30% of sales tonnage. EAF-only SCM-grade availability is not separately reported [1]. Despite representing a smaller share by tonnage, GGBFS contributed roughly 80% of the $620 million in total slag sales, with electric arc furnace (EAF) and basic oxygen furnace (BOF) slags making up the remainder. Production levels have remained relatively consistent over the past five years, though regional supply dynamics vary considerably [71].

Recent laboratory studies indicate that processed or carbonated EAF slags can act as partial cement replacements or supplementary cementitious materials in blended systems, with acceptable strength and durability in some cases, although performance remains highly dependent on slag composition, cooling history, and activation route [64,69,74]. Taken together, these results position EAF slag as an emerging cementitious resource with substantial steelmaking-derived feedstock potential, but significant technical, variability, and specification barriers that currently confine its use primarily to aggregate applications [75,76,77], with broader adoption and qualification issues considered later in this review.

### 4.4. Waste Glass

Waste glass presents significant potential as an SCM due to its high silica content, typically about 70 to 75% SiO_2_, and its amorphous structure, which can provide pozzolanic reactivity when ground to appropriate fineness [78,79]. Glass waste is generated from various streams including container glass, flat glass from construction/automotive sources, and specialty glass products. Processing involves collection, sorting by color and type, cleaning to remove contaminants, and fine grinding to achieve particle sizes comparable to or finer than cement for optimal reactivity [78]. In 2018, the United States generated 12.25 million short tons of municipal-waste glass, of which 3.06 million tons were recycled (~25%) [80]. While this volume appears substantial in aggregate, only the finely ground, contaminant-free fraction of collected glass is suitable for use as an SCM. The coarse or mixed-color glass streams, which constitute a significant share of total waste generation, require additional sorting, cleaning, and grinding investment before they can contribute to cementitious applications, meaning the realistically usable SCM-grade fraction is considerably smaller than total waste glass generation.

Major sources include municipal solid waste streams and construction/demolition debris. Primary drawbacks for its expanded reutilization include the energy-intensive grinding required to achieve sufficient fineness for pozzolanic activity, soluble alkali content when ground, variability in composition based on glass type and color, and the need for effective contaminant removal. Potential alkali-silica reaction (ASR) is a separate and well-documented concern for coarse or unground glass used as aggregate, rather than the finely ground, pozzolanic-grade material discussed in this section. The ASR risk for finely ground glass powder is generally low, and, in some studies, ground glass has been shown to mitigate ASR expansion through pozzolanic consumption of reactive silica [81]. Practical deployment must therefore address both the logistics of collecting relatively clean glass fractions and the cost of fine grinding, contaminant removal, and quality control [82,83,84]. Regional availability varies significantly based on waste management infrastructure and glass recycling operations [85]. Even with these drawbacks, finely ground waste glass is a technically promising but still emerging cementitious resource, with performance, durability, and specification issues that need further resolution before broad, mainstream use as an SCM [78,85,86].

### 4.5. Carbonated Binders

While concrete naturally absorbs atmospheric CO_2_ over its service life through ambient carbonation, recent thermodynamic and diffusion-based modeling indicates that this passive process is a trivial contributor to offsetting cement-production emissions. Xiao et al. report that by 2030, the ambient carbonation of concrete placed in service globally is projected to uptake approximately 0.23 gigatonnes of CO_2_ annually, less than 10% of annual cement clinker emissions and that a fully exposed structural concrete element would require up to 1000 years to reach 50% carbonation under ambient conditions [87]. Complementary life-cycle assessments of engineered CO_2_ mineralization technologies further indicate that only a subset of currently deployed approaches are both economically competitive and capable of meaningfully reducing CO_2_-equivalent emissions at scale, with the most promising pathways relying on intentional carbonation of end-of-life cement paste, recycled concrete fines, and other materials amenable to carbonation rather than passive exposure of in-service concrete [88].

Carbonation curing applies controlled exposure of fresh cement-based mixtures to CO_2_ during early curing to accelerate strength development and convert the absorbed CO_2_ into calcium carbonate and related phases, partially replacing conventional cementitious reactions while relying on familiar cementitious feedstocks rather than entirely new clinker chemistries [89,90]. This route is commonly applied in precast production, including blocks and pavers, where reaction conditions can be tightly controlled and flue gas or purified CO_2_ streams are available [89].

A related but distinct route applies carbonation to end-of-life cementitious material, including recycled cement paste powders and recycled concrete fines or aggregates, rather than fresh mixtures during curing [91]. Because this material is already hydrated, carbonation instead targets residual calcium-rich phases, converting them into calcium carbonate to refine microstructure, reduce porosity, and improve reactivity and filler value for reuse in new binder systems. This treatment route is one specific application of the broader concept of CO_2_ mineralization, which describes the deliberate conversion of CO_2_ into stable carbonate phases across cementitious and non-cementitious feedstocks alike, and is not limited to recycled concrete-derived materials [89].

From a material and performance perspective, accelerated carbonation of cement pastes and recycled cementitious materials can alter phase assemblages, microstructure, and engineering properties in ways that differ from conventional hydration [90,92]. Studies of wet and gas-phase carbonation of cement paste and recycled fines report the formation of calcite and other carbonate phases, changes in the calcium silicate hydrate structure, and reductions in porosity and water absorption under optimized curing conditions [93,94]. In carbonation curing systems specifically, this process enhances early-age strength and refines pore structure, leading to improved mechanical performance and transport properties compared with reference mixtures cured under standard conditions [89,95]. In one study, recycled cement paste powder achieved approximately 19% CO_2_ uptake after 2 h of carbonation at 80% CO_2_ [94]. In a separate study, 10–20% replacement with carbonated hardened cement paste powder maintained or improved strength, while 30% replacement caused a slight reduction [96].

Despite this technical progress, carbonated binders remain at an emerging stage of deployment. Practical implementation requires reliable CO_2_ supply, gas handling and safety systems, process control over temperature, humidity, and exposure duration, and integration with existing plant operations [89,97]. Reaction kinetics, scale-up models, and long-term durability under field conditions still need further validation, particularly for systems that rely on recycled cementitious feedstocks and complex waste streams [91,93,95]. Qualification pathways remain less established for carbonated recycled powders used as SCMs and will require application-specific performance and durability data. For these reasons, carbonation-cured binders and CO_2_-mineralized concrete are best viewed as an emerging binder strategy built on existing cementitious materials, with substantial potential for performance gains through carbonated materials but significant infrastructure, process, and qualification hurdles that must be addressed before widespread adoption.

### 4.6. Biocementation

Unlike the other material families discussed in this review, biocementation is included here not as a candidate for direct, large-volume replacement of Portland cement in conventional concrete, but as a longer-term and mechanistically distinct alternative cementation pathway whose current applications remain concentrated in soil stabilization, erosion control, dust suppression, and related geotechnical uses rather than structural concrete production.

Biocementation employs biological processes to precipitate calcium carbonate or other minerals, creating cemented materials through microbial-induced calcite precipitation (MICP) or enzyme-induced carbonate precipitation (EICP) [98,99]. In MICP, ureolytic bacteria such as Sporosarcina pasteurii hydrolyze urea in the presence of calcium ions, raising local pH and precipitating CaCO_3_ crystals that bind particles [100]. EICP uses purified urease enzyme rather than live bacteria, which can offer improved process control, storage stability, and suitability for fine-grained media where bacterial transport is limited [101]. Alternative systems employ other bacteria or enzymes that catalyze different carbonate precipitation reactions or use non-ureolytic pathways to avoid ammonia generation [102,103]. Feedstocks typically include bacterial cultures or enzyme solutions, urea or alternative substrates, calcium sources such as CaCl_2_ solution, and the material to be cemented, most often sand, aggregate, or soil [98,104]. Cementation occurs through multiple treatment cycles in which microbial or enzyme solutions and cementation solutions are applied sequentially or simultaneously, allowing CaCO_3_ precipitation to progressively fill pores and bind grains [105].

Achieved strengths and stiffness vary widely based on treatment regime and application. In one MICP sand study, percolation treatment produced UCS values of approximately 0.5–0.9 MPa, while a combined treatment method achieved 3.7 MPa [104]. Laboratory specimens of cemented sands and granular materials have achieved strengths in the range needed for niche construction or barrier applications, and recent work has explored biocementation for enhancing the performance of recycled aggregate concrete by precipitating CaCO_3_ at aggregate surfaces and in interfacial transition zones [106,107,108]. Technology readiness remains at pilot or demonstration scale for geotechnical applications. Biological scale-up has been demonstrated using 900 L culture batches and a 1.5 m, 650 kg sand column [109], while structural concrete replacement and large-volume binder applications remain largely conceptual [98,99,109].

Key barriers include high cost of reagents, particularly enzyme-grade urease and nutrient solutions; scalability challenges for large-volume applications that require controlled delivery of microbes or enzymes and reactants; and by-product generation such as ammonia from urea hydrolysis, which raises environmental and handling concerns [99,100,101]. Limited durability data under realistic service conditions, relatively slow cementation rates compared to conventional clinker-based materials, and the lack of established quality control protocols and design standards further constrain near-term deployment [110]. Most current development therefore focuses on geotechnical and niche applications such as ground improvement, dust suppression, erosion control, and crack healing, rather than direct structural concrete replacement. For this review, biocementation is best viewed as an emerging binder concept with promising low-carbon potential and diverse application avenues, but with substantial biological, process, and regulatory challenges to be addressed before broader use in mainstream cementitious systems.

## 5. Information Needed for Adoption

Broad deployment of the material families discussed in Section 3 and Section 4 depends on more than technical feasibility alone. Even materials with well-documented pozzolanic or hydraulic behavior require standardized characterization methods, durability evidence under service conditions, clear specification pathways, and reliable supply logistics before they can be routinely specified by owners and agencies. This section synthesizes these cross-cutting requirements, which were deliberately separated from the material-specific discussions in Section 3 and Section 4 so that common themes could be compared directly across material families.

**Figure 2:**
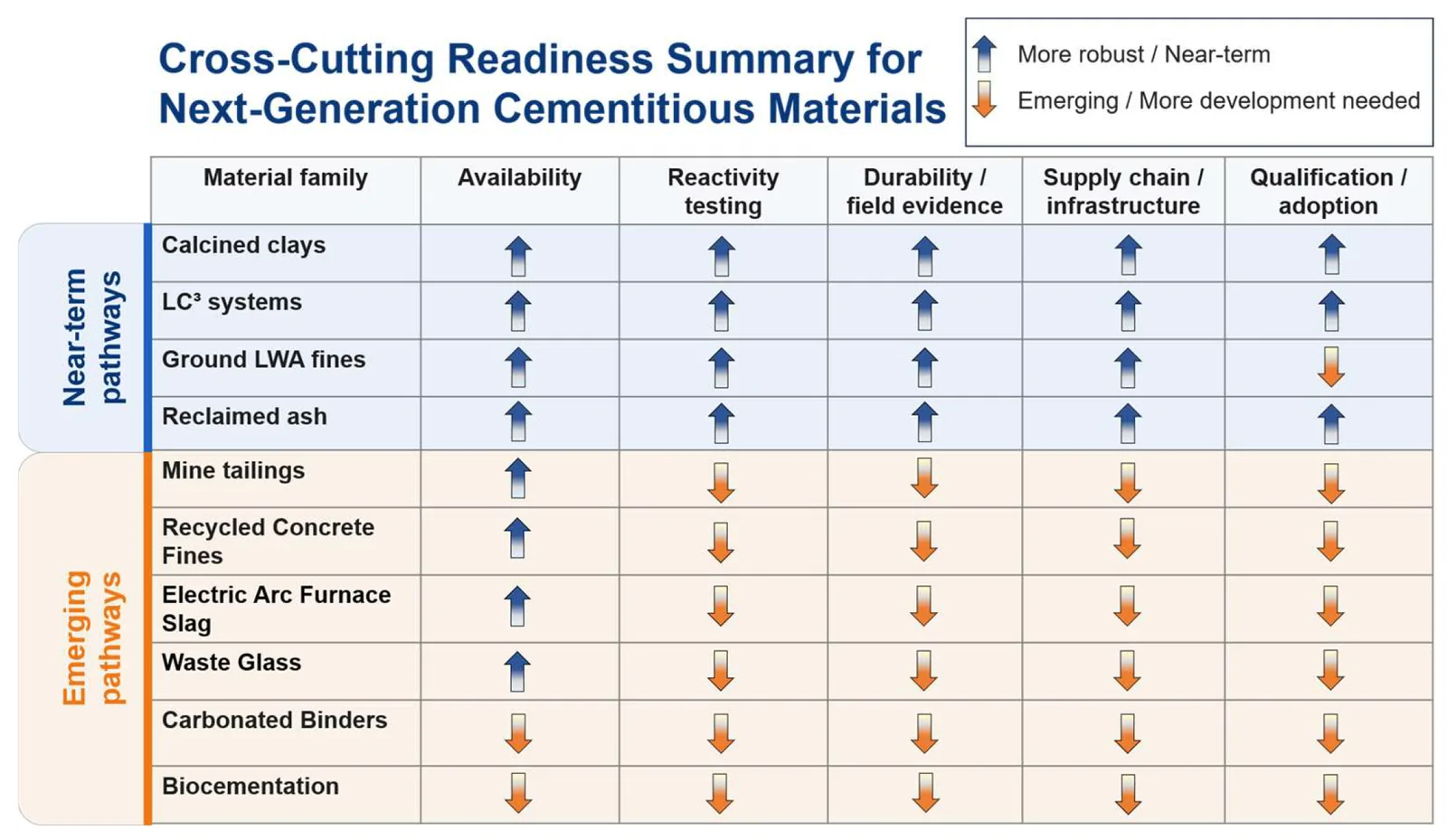
Qualitative readiness of next-generation cementitious materials across availability, reactivity testing, durability and field evidence, supply-chain infrastructure, and qualification and adoption. Upward arrows indicate comparatively robust or near-term status, whereas downward arrows indicate emerging status requiring additional development. Supporting quantitative data and references for each material family are summarized in Table 1.

Table 1 consolidates the quantitative findings from Section 3 and Section 4 and highlights several patterns. First, reported feedstock availability does not necessarily correspond to deployment readiness. More than 8 Gt of mine tailings were generated globally in 2016, while approximately 16 Mt of total iron and steel slag were sold in the United States in 2025; however, the fraction of these materials suitable for use as SCMs remains source-specific and depends on processing, characterization, and qualification. In contrast, calcined clays and reclaimed ash have clearer near-term pathways supported by commercial production or harvesting and existing standards or guidance. Second, reported replacement levels vary substantially among material families and should not be directly compared because studies evaluate different material roles, including cement replacement, blended SCM systems, and aggregate substitution. Third, many emerging materials require processing such as fine grinding, mechanochemical activation, or carbonation to achieve the reported performance. Ground-glass pozzolan is covered by ASTM C1866/C1866M, while other emerging materials may require source- and application-specific evaluation using approaches such as ASTM C1709. These quantitative trends underpin the readiness classification summarized qualitatively in Figure 2.

The successful transition to next-generation cementitious materials requires moving beyond traditional prescriptive specifications toward performance-based frameworks that evaluate materials based on their functional contribution to concrete properties rather than their chemical composition alone. Central to this transition is the need for rapid, reliable methods to assess SCM reactivity. The traditional strength activity index (SAI) test (ASTM C311) [115] requires up to 28 days and reflects mortar performance rather than isolating intrinsic SCM reactivity, limiting its usefulness as a rapid screening method for diverse alternative materials [116].

### 5.1. Reactivity Testing and Characterization

The recent standardization of the R3 test (ASTM C1897, Standard Test Methods for Measuring the Reactivity of Supplementary Cementitious Materials by Isothermal Calorimetry and Bound Water Measurements) represents a significant advancement [111]. Developed through RILEM Technical Committee 267-TRM, the R^3^ test measures the heat release or bound water of an SCM in a simplified model system containing calcium hydroxide, calcium carbonate, potassium sulfate, potassium hydroxide, SCM, and water at 40 °C for 7 days. This provides a reliable, rapid proxy for both pozzolanic and latent hydraulic reactivity, bypassing the lengthy mortar-based SAI tests. Interlaboratory studies have demonstrated good reproducibility, and correlations between R3 heat values and 28 day compressive strength in blended cements have been validated across a wide range of material types including fly ash, slag, calcined clays, natural pozzolans, and alternative SCMs [29,117].

### 5.2. Long-Term Durability and Field Performance Evidence

A recurring gap across nearly every material family reviewed here is the scarcity of long-term, field-scale durability data. Laboratory studies on calcined clays, LC3, mine tailings, recycled concrete fines, EAF slag, waste glass, and biocementation concepts consistently report favorable 28-day or 90-day mechanical performance, but far fewer studies extend to multi-year exposure under realistic freeze–thaw, chloride, sulfate, or carbonation conditions typical of U.S. infrastructure service life [118]. This asymmetry between short-term laboratory promise and long-term field validation is a primary reason many of these materials remain classified as emerging rather than near-term, even when their fundamental chemistry is well understood.

Closing this gap requires coordinated, multi-year field demonstration programs, ideally located across different U.S. climate regions, and instrumented to track chloride ingress, expansion, cracking, and strength development over 5- to 10-year horizons rather than standard 28- or 56-day windows [118,119]. State DOT research and qualification programs evaluating alternative SCMs provide a valuable source of performance data that can support broader adoption [119]. For emerging materials such as mine tailings and EAF slag, where compositional variability is extreme, durability evidence must also be tied to specific feedstock sources and activation methods rather than being treated as a single generic material behavior.

### 5.3. Supply-Chain and Infrastructure Considerations

Beyond technical performance, the feasibility of scaling any material family depends on the logistics of collection, processing, and distribution relative to concrete demand centers. Near-term materials such as reclaimed ash and calcined clays benefit from existing collection, beneficiation, and grinding infrastructure that can be adapted with incremental investment, which is a major reason they are positioned ahead of emerging materials in this review’s readiness framework. In contrast, emerging materials such as mine tailings and recycled concrete fines face a more fundamental infrastructure gap: tailings are often located far from concrete markets and require new aggregation and transportation networks, while recycled concrete fines depend on carbonation processing capacity that does not yet exist at commercial scale in most U.S. regions.

Waste glass streams add a further complication, since their generation is distributed across many small municipal and industrial sources rather than concentrated at a few large facilities, as is the case with coal ash or steel slag. This distributed generation pattern raises aggregation costs and complicates quality control, since material from many small sources must be blended or characterized individually. For EAF slag specifically, the ongoing structural shift in U.S. steel production toward electric arc furnaces means that supply volumes are likely to grow even as current beneficiation limitations continue to constrain the amount of usable SCM-grade material, making near-term investment in beneficiation capacity especially urgent. Addressing these supply-chain gaps will likely require targeted public or industry investment in regional processing hubs, particularly for materials whose largest volumes are geographically distant from major concrete production and construction markets.

### 5.4. Qualification Pathways and Standards

ASTM C1709, Standard Guide for Evaluation of Alternative Supplementary Cementitious Materials for Use in Concrete, provides a technical approach for source-specific evaluation of alternative SCMs that fall outside established prescriptive specifications. Testing is selected based on the characteristics of the material and its intended concrete application. ASTM C1709 can support evaluation of alternative SCMs, but broader acceptance also depends on field-performance evidence, applicable agency requirements, and development of appropriate material- or project-specific specification [112]. This challenge is consistent with recent market analysis indicating that many next-generation cement and concrete technologies face a pronounced capitalization gap after an initial funding round, where standards compatibility, verified field performance, and workforce readiness, rather than technology readiness alone, determine the pace of commercial deployment [120].

Figure 2 synthesizes the relative readiness of each material family across availability, technical performance (from Section 3 and Section 4), and the cross-cutting reactivity, durability, supply-chain, and qualification requirements discussed in Section 5. Taken together, these five elements, availability, reactivity testing, durability and field validation, supply-chain readiness, and formal qualification pathways, form an interdependent sequence rather than a checklist of independent requirements.

Rapid reactivity testing under ASTM C1897 can support initial screening of materials evaluated using ASTM C1709, but it cannot substitute for field-performance evidence, particularly for materials with high compositional variability such as mine tailings and EAF slag. Similarly, even a material with excellent laboratory reactivity and durability data will struggle to reach commercial specification if aggregation and processing infrastructure is not developed in parallel, as is currently the case for recycled concrete fines and waste glass. The near-term versus emerging classification used throughout this review reflects, in large part, how far each material family has progressed along this combined technical and infrastructural pathway. Closing the remaining gaps will require coordinated investment from researchers, standards bodies, and major buyers, an agenda outlined in the concluding section of this review.

## 6. Conclusions

This review has synthesized the current landscape of next-generation cementitious materials available to the U.S. concrete industry through approximately 2035–2040, organized around a two-tier readiness framework. Calcined clays, LC3-type systems, ground lightweight aggregate fines, and reclaimed ash are closest to broader deployment, supported by existing production infrastructure and partial standards coverage. Mine tailings, recycled concrete fines, electric arc furnace slag, waste glass, carbonated binders, and biocementation concepts remain emerging, limited less by technical promise than by compositional variability, immature processing infrastructure, and uneven or application-specific qualification pathways.

Across both tiers, availability alone does not guarantee deployment: materials with large feedstock volumes but weak qualification pathways, such as mine tailings and EAF slag, may advance more slowly than lower-volume materials, such as calcined clays and LWA, that can build on existing infrastructure. This confirms that reactivity testing, long-term durability evidence, supply-chain readiness, and formal qualification pathways function as an interdependent sequence, rather than independent, substitutable requirements.

For researchers, extending rapid reactivity testing protocols such as ASTM C1897 to compositionally variable emerging materials, and pursuing multi-year field durability studies, represent the most direct opportunities to close current evidence gaps. For standards bodies, advancing evaluation and qualification frameworks such as ASTM C1709 and developing dedicated classification criteria for materials such as LWA, mine tailings, and EAF slag would allow performance-based specification to better accommodate compositional variability. For major buyers and specifying agencies, aggregating and sharing field-performance data from existing research, qualification programs, and pilot projects offers an underutilized route to shortening qualification timelines elsewhere.

The cement and concrete industry can no longer assume stable, high-quality SCM supply from coal fly ash and blast furnace slag. While no single material family reviewed here replaces that legacy supply, together they offer a diversified portfolio of near-term and longer-horizon pathways whose realization will depend as much on coordinated technical, regulatory, and logistical progress as on further proof-of-concept research.

## Figures and Tables

**Table 1:** Summary of reported availability, replacement levels, quantitative performance, development scale, limitations, and standards status for the cementitious material families reviewed in Section 3 and Section 4.

	Availability (Reported)	Replacement (% of Cement)	Key Quantitative Findings	Scale	Main Limitations	Standard/Qualification Route
**Calcined clays (near-term)**	Widespread clay deposits; 4 commercial U.S. calcination plants (as of May 2025) [14]	5–20 [26,27]	Reactive after calcination at ~650–850 °C; improved early-age strength, lower permeability, ASR mitigation [26,27]	Commercial production in the U.S. [14]	Reactivity depends on kaolinite content and calcination conditions [28,29,30]	ASTM C1945-26b; historically Class N under ASTM C618; [37,38]; R3 reactivity measured using, ASTM C1897 [111]
**LC^3^ systems (near-term)**	Clay and limestone widely available; U.S. LC^3^ production remains limited, with several announced or developing projects [16,17,18]	Common LC^3^-50 formulations use ~50% clinker, with ~45% calcined clay + limestone and ~5% gypsum [15]	LC^3^ blends with ~50% clinker can achieve strength comparable to or greater than OPC at equal w/b, particularly at later ages, with reduced chloride ingress [15,31]	Demonstration/early commercial; several U.S. projects have been announced or are under development [16,17,18]	Sulfate and alkali balance, workability, and product- or agency-specific qualification requirements [15]	Existing U.S. standards cover some calcined-clay and blended-cement use, but current limits do not fully accommodate typical LC^3^ replacement levels; broader agency adoption remains limited [18]
**Ground LWA (near-term)**	Expanded shale, clay, and slate LWA is produced commercially in the U.S.; ground fines are a by-product stream whose recoverable volume is not well quantified [21,22]	About 10–20% has been studied; ~15% is a representative reported level [23,33]	At 15% cement replacement, 28-day strength was similar to control at 20 °C, ~15% higher at 35 °C, and 10–15% lower at 5 °C [33]	Laboratory; in-plant by-product use [24,33]	Reactivity depends strongly on fineness and firing history; fines are not currently a dedicated SCM stream [24]	No dedicated material specification identified; broader alternative-SCM qualification approaches may apply.
**Reclaimed ash (near-term)**	20.2 million short tons of fly ash disposed in 2017 at 179 U.S. utility plants; >1 billion tons of legacy fly ash stored in landfills and surface impoundments [5,41].	15–25% Class F; 20–35% Class C; 50–70% in high-volume fly ash mixtures with high-range water reducer; reclaimed ash requires source-specific qualification [36].	Site-specific beneficiation can produce harvested fly ash meeting ASTM requirements for concrete use [41,42].	Large-scale commercial harvesting supplying concrete markets in South Carolina and Pennsylvania [5,42].	Site-to-site variability, beneficiation energy and cost, contaminants, and source-specific qualification [41,44].	ASTM C618; ASTM E3183-24 for harvesting guidance [37,43].
**Mine tailings (emerging)**	>8 Gt of tailings generated globally in 2016; ~223 Gt stored in ~29,000–35,000 active, inactive, and abandoned facilities globally; U.S. totals remain uncertain [45,46].	20% activated Pb–Zn tailings; 30% iron-tailings/FA/GGBFS SCM blend; 30% mechanically activated tailings evaluated in paste [48,49,50].	30% iron-tailings/FA/GGBFS SCM blend reached 49 MPa at 28 d; 20% activated Pb–Zn tailings reached 40.85 MPa at 90 d vs. 38.15 MPa control [49,50].	Laboratory [48,49,50].	Strong source-to-source variability; sulfides and trace metals; remote locations and transportation; mechanical/thermal activation may add energy demand [51,52,53].	ASTM C1709, source-specific evaluation [112]
**Recycled concrete fines (emerging)**	Fine fraction (<150 µm) accounts for ~20–30% of waste concrete generated during recycled-aggregate production [57]	Cement replacement studied up to ~30%; performance and workable dosage depend on source, treatment, and admixture use [55,56]	Aggregate-use example: at 70% sand replacement, carbonated fines gave ~20% higher 28-d mortar strength than non-carbonated fines [59]	Laboratory [55,56,59]	Source variability, high water demand, contaminants, and need for dedicated carbonation processing and source-specific quality control [55,56,57]	ASTM C1709, source-specific evaluation [112]
**EAF slag (emerging)**	~16 Mt of total iron and steel slag sold in the U.S. in 2025; granulated blast-furnace slag accounted for <30% of sales tonnage; EAF-only SCM-grade availability is not separately reported [1].	10–30% evaluated as cement replacement [64]	At 30% replacement, carbonated EAF slag containing 8.6% CO_2_ developed higher 28-d strength than corresponding as-received and hydrated EAF-slag mortars; early hydration was delayed [64].	Laboratory cement-replacement studies; broader EAF-slag carbonation and utilization under development [64,65,74]	Crystalline phases and low inherent reactivity; free CaO/MgO can cause volume instability; processing needed to improve SCM performance [65,67,68]	ASTM C1709-22, source-specific evaluation; EAF slag is currently used primarily in aggregate applications [75,76,77,112]
**Waste glass, ground (emerging)**	12.25 million short tons of U.S. municipal-waste glass generated in 2018; 3.06 million tons recycled (~25%) [80]	5–40% studied; 30% evaluated in long-term mortar studies [78,86]	At 30%: 80–86% of control strength at 28 d and up to 108% activity index at 6 months; at 30% GP, strength was 45% below control at 3 d and 16% below at 90 d; 30–40% GP reduced autoclave expansion by up to 50% [78,86]	Laboratory; regional municipal/DOT projects demonstrated [113]	Grinding energy, glass composition/alkali content, sorting, and contamination [82,83]	ASTM C1866/1866M-25 [114]
**Carbonated binders (emerging)**	Depends on availability of concentrated CO_2_ and Ca-rich recycled cementitious feedstocks [89,91]	10–20% carbonated hardened cement paste powder maintained or improved strength; 30% caused a slight strength reduction in one study [96]	~19% CO_2_ uptake after 2 h carbonation of recycled cement paste powder at 80% CO_2_ in one study; uptake is strongly process- and feedstock-dependent [94]	Laboratory; Fresh-concrete carbonation: TRL 9; carbonation-cured precast products: TRL 8; direct carbonation of recycled cement paste for SCM use: TRL ~4 [89]	CO_2_ supply and handling, process control, transport/logistics of recycled fines, scale-up, and durability/application-specific performance [89,91,95]	No dedicated specification identified for carbonated recycled cement paste powder used as an SCM; qualification is application- and product-specific.
**Biocementation, MICP/EICP (emerging)**	Reagent-based: urea or alternative substrate, Ca^2+^ source, and ureolytic bacteria or urease enzyme [98,101]	Not applicable; primarily soil/sand cementation rather than cement replacement [98,101]	MICP-treated sand achieved 0.5–0.9 MPa UCS by percolation and 3.7 MPa using a combined treatment method; scale-up demonstrated 900 L culture batches and a 1.5 m, 650 kg sand column [104,109]	Laboratory to pilot/field-oriented geotechnical demonstrations; microbial culture scaled to 900 L batches with continuous cultivation totaling 5400 L [98,99,109]	Reagent/enzyme cost, ammonia generation for ureolytic routes, treatment time, delivery/scale-up, durability, and limited standardized QC/design procedures [99,100,101,110]	No dedicated mainstream structural-concrete binder specification identified; project-specific geotechnical evaluation required

Notes: Reported availability values represent published production, sales, disposal, or inventory estimates and should not be interpreted as deployable SCM-grade supply. Replacement percentages are by mass of cement unless otherwise noted; LC^3^ values describe blend composition rather than cement replacement, and the recycled-concrete-fines strength example is based on sand replacement. Quantitative performance values are study-specific and depend on feedstock, processing, mixture proportions, curing, and test conditions. Development scale reflects the evidence reviewed and does not imply a formal technology-readiness level unless explicitly stated. ASTM C1709 provides guidance for source-specific evaluation of alternative SCMs and does not itself constitute certification or agency approval. Units are reported as given in the cited sources; Mt = million metric tonnes and Gt = billion metric tonnes, while short tons are identified explicitly. Abbreviations: AAR, alkali-aggregate reaction; ASR, alkali–silica reaction; C&D, construction and demolition; EAF, electric arc furnace; EICP, enzyme-induced carbonate precipitation; FA, fly ash; GGBFS, ground-granulated blast-furnace slag; GP, glass powder; LC^3^, limestone calcined clay cement; LOI, loss on ignition; LWA, lightweight aggregate; MICP, microbial-induced calcite precipitation; OPC, ordinary Portland cement; SAI, strength activity index; SCM, supplementary cementitious material; SP, superplasticizer; TRL, technology readiness level; UCS, unconfined compressive strength; w/b, water-to-binder ratio.

## Data Availability

No new data were created or analyzed in this study. Data sharing is not applicable to this article.
